# Anti-leptospirosis agglutinins in Brazilian capybaras (*hydrochoerus hydrochaeris*)

**DOI:** 10.1186/s40409-016-0059-6

**Published:** 2016-01-27

**Authors:** Helio Langoni, Ivone Yumi Kuribara, Ana Paula Ferreira Lopes Correa, Leila Sabrina Ullmann, Gabriela Pacheco Sánchez, Simone Baldini Lucheis

**Affiliations:** Department of Veterinary Hygiene and Public Health, School of Veterinary Medicine and Animal Husbandry, São Paulo State University (UNESP – Univ Estadual Paulista), Botucatu, SP Brazil; São Paulo Agency of Agribusiness Technology (APTA/SAA), Bauru, São Paulo State Brazil; Departamento de Higiene Veterinária e Saúde Pública, Faculdade de Medicina Veterinária e Zootecnia, UNESP, Distrito de Rubião Jr, s/n, Botucatu, SP 18618-000 Brazil

**Keywords:** Leptospirosis, Capybaras, *Hydrochoerus hydrochaeris*, Serologic evaluation, Zoonosis

## Abstract

**Background:**

The interest in commercial use of wild animals is increasing, especially regarding raising of capybaras. Although this wild species is potentially lucrative for the production of meat, oil and leather, it is suggested as a probable reservoir of leptospires.

**Methods:**

Due to the economic importance of this species and the lack of studies concerning leptospirosis, the presence of anti-leptospirosis agglutinins was assayed in 55 serum samples of capybaras (*Hydrochoerus hydrochaeris*) from commercial and experimental breeding flocks located in São Paulo state, Paraná state, and Rio Grande do Sul state, Brazil. Samples were obtained through cephalic or femoral venipunction (5 to 10 mL). Microscopic agglutination test was used according to the Brazilian Health Ministry considering as cut-off titer of 100.

**Results:**

Out of the 55 samples analyzed, 23 (41.82 %) tested positive. The most prevalent serovar was Icterohaemorrhagiae (56.52 %) in 13 samples, followed by Copenhageni in nine samples (39.13 %), Pomona in four samples (17.39 %), Djasiman and Castellonis in three samples each (13.04 %), Grippotyphosa, Hardjo, Canicola, and Cynopteri in two samples each (8.7 %), and Andamana and Bratislava in one sample each (4.34 %).

**Conclusions:**

These results suggest the evidence of exposure to *Leptospira* spp. and the need of new studies to evaluate a higher number of capybaras from different regions to better understand the importance of leptospirosis infection in these animals and verify the zoonotic role of this species as a possible source of infection to humans and other animals.

## Background

Leptospirosis is a bacterial disease that causes morbidity and mortality around the world, except in Antarctica [1–3]. Although it is endemic in many rural and urban communities and can also cause sporadic epidemics, little is actually known about the true disease burden [4, 5]. Its lethality can achieve 40 % in acute human cases with more than 500 thousand cases of the severe form being reported annually worldwide [6, 7].

The disease is caused by a bacterium of the order Spirochetales, family Leptospiraceae, and genus *Leptospira* [4, 8]. In 2007, taxonomy was reformulated and *Leptospira* was divided into 13 pathogenic species (*L. alexanderi*, *L. alstonii*, *L. borgpetersenii*, *L. inadai*, *L. interrogans*, *L. fainei*, *L. kirschneri*, *L. licerasiae*, *L. noguchi*, *L. santarosai*, *L. terpstrae*, *L. wielli* and *L. wolffii*) and six saphrophytic (*L. biflexa*, *L. meyeri*, *L. yanagawae*, *L. kmetyi*, *L. vanthielii* and *L. wolbachii*) [1, 9].

*Leptospira* species are classified in serogroups composed by more than 260 serovars based on antigenic characteristics by microscopic agglutination. Serotyping has been recognized as an essential tool in clinic and epidemiological investigations and it can indicate the reservoir involvement in the disease transmission [10–12].

Leptospirosis can be transmitted directly through contact with secretions, blood or urine of infected animals or indirectly through the contact with water contaminated mainly with urine of carriers [13]. Many domestic and wild animals get infected and become renal carriers and potential shedders of the agent [5, 14, 15]. Evidence of renal *Leptospira* spp. carriers has been found in mammals, and this fact is a central component on the persistence and epidemiology of leptospirosis [1].

Its occurrence is related to precarious conditions of sanitary infrastructure and high rodent infestation, which are considered universal *Leptospira* reservoirs. Flooding allows the dissemination and persistence of the causal agent in the environment, facilitating outbreak occurrence [16].

Many wild animals, among them rodents, are adapted to *Leptospira* and they do not show clinical signs or lesions [17]. Rodents are considered the main reservoirs of the pathogen because they harbor *Leptospira* in their kidneys and eliminate them actively in their urine into the environment, contaminating water, soil and food. They are particularly important due to their cosmopolitan distribution and have been responsible for economic and sanitary damages caused to humans [2, 16].

Rodents compose the biggest order within Mammalia class. In South America, these animals are more numerous in species and abundance than in other continents. Capybaras (*Hydrochaeris hydrochaeris*) are the biggest herbivorous rodents worldwide and inhabit all Brazilian states with high zootechnical potential due to their large size, feed conversion, prolificacy, rusticity, and the habit of nursing their puppies until the beginning of reproduction [18, 19]. Due to leptospirosis occupational character and the fact that rodents are considered universal *Leptospira* reservoirs, breeding with zootechnical purposes may favor the disease transmission in nurseries without adequate zoosanitary management.

In nurseries for capybaras with zootechnical purposes and adequate environment and feeding, the occurrence of diseases is low and wounds due to fights and parasitic diseases in general are the most common. The occurrence of abortion in diverse pregnancy phases is relatively common but the cause is unknown [20].

The first description of leptospirosis in capybaras was carried out in animals from a commercial slaughter and 178 samples were tested by microscopic agglutination test. From those, 111 (63.3 %) were reagent to several serovars and the most prevalent were Canicola, Hardjo, and Wolfii. Renal tissue culture was also performed, which confirmed the occurrence of Canicola serovar [21].

Bananal serovar is an autochotonous Brazilian strain included in Grippothyphosa serogroup that was isolated from kidney of capybaras slaughtered in Piracicaba, São Paulo state, Brazil [22]. In fact, serovar Bananal was first isolated from capybaras in Brazil, but has also been found in other wild rodents and goats, indicating the dissemination of this serovar among multiple species, favoring the transmission of the disease [23].

Another study evaluated three wild capybaras by microscopic agglutination and one was reagent to serovar Patoc [24]. In a study carried out with captive animals, five out of 12 were seropositive to serovar Hardjo by microscopic agglutination [25].

Among 22 capybaras slaughtered in Rio Grande do Sul, four animals were reagent to Australis serogroup (Bratislava and Australis serogroup), with titers varying from 100 to 3200, and two animals were positive to *Leptospira illini*, a saprophyte spirochaeta phylogenetically close to leptospires. This situation indicates that nurseries for captive capybaras may act as pathogenic *Leptospira* reservoirs, which demands attention concerning occupational risks involved in breeding and slaughtering this species [26].

There is scarce information on the role of capybaras in leptospirosis, as a source of infection or reservoir to the pathogen. In an experimental infection study, *Leptospira* serovar Pomona was inoculated and seroconversion, leptospiremia and leptospiruria were evaluated in seven capybaras. Agglutinins were detected between two and seven days after inoculation with titer peak (3200) between days 9 and 27, persisting until 83 days after inoculation. Leptospiremia was confirmed in five animals and leptospiruria in four between days 9 and 42 after inoculation. Isolation and leptospire detection were negative from kidney and liver fragments. Authors concluded that capybaras are susceptible to leptospirosis and show seroconversion, leptospiremia and leptospiruria, characteristics similar to other species and may act as source of infection to humans and other animals [18].

Leptospirosis is important among occupational diseases and in public health. Additionally, it may cause important economic losses in livestock. Thus, the aim of the present study was to isolate anti-leptospirosis antibodies in capybaras to perform a serological survey.

## Methods

The study was conducted in five distinct regions from three distinct states: Guararapes and Piracicaba in São Paulo state, Curitiba and Foz do Iguaçu in Paraná state, and Sapucaia do Sul in Rio Grande do Sul state, Brazil. Fifty-five capybaras (*Hydrochaeris hydrochaeris*) from both genders and from commercial and experimental breeding facilities were studied. Animals were sedated by using midazolam and ketamine according to the dosages indicated for blood collection and serum separation [27].

Samples were tested by microscopic agglutination using as antigens standard strains of *Leptospira* maintained to weekly subcultures in EMJH liquid media at the Zoonosis Research Center (NUPEZO). Serovars tested included Andamana, Australis, Autumnalis, Bratislava, Castellonis, Canicola, Cynopteri, Copenhageni, Djasiman, Grippotyphosa, Hardjo, Icterohaemorrhagiae, Pyrogenes, Pomona, Tarassovi and Wolffi. The animals were considered reactive when they presented titers greater than or equal to 100. The final titer was the one that presented an agglutination rate at least 50 % according to Faine [2].

## Results

Out of the 55 samples analyzed, 23 (41.82 %) were reagent and the most prevalent serovar was Icterohaemorrhagiae (56.52 %) in 12 samples from Sapucaia do Sul and one from Guararapes, followed by Copenhageni in nine samples (39.13 %), Pomona in four samples (17.39 %), Djasiman and Castellonis in three samples each (13.04 %), Gryppotyphosa, Hardjo, Canicola and Cynopteri in two samples each (8.7 %), and Andamana and Bratislava in one sample each (4.34 %). The highest titer was 400 and the most prevalent titer was 100.

## Discussion

The results obtained in this study show that capybaras may be infected with different *Leptospira* spp. Serovars. However, it is not possible in this case to prove the transmission risk for others animals and humans, because only the serological response in animals was studied.

It can be observed that the most prevalent serovar was Icterohaemorrhagiae, which is important to public health since *Rattus norvegicus* is its main host, indicating environment contamination from urine of these animals. Copenhageni was also prevalent in this study, which does not agree with findings by Nishiyama et al. [25] that demonstrated that among 12 studied capybaras, five were reagent only to serovar Hardjo, more prevalent in bovines. Silva et al. [28], in Rio Grande do Sul, Brazil, reported that four out of 22 animals were reagent to serovar Bratislava (more prevalent in swine and dogs) and Australis (mostly found in dogs). Chiacchio et al. [29] also found high titers (˃ 1800) for serovars Grippotyphosa, Hardjo and Pomona in 31 wild capybaras from São Paulo, SP, Brazil. Paixão et al. [30] verified the occurrence of *Leptospira* spp. among rats captured in a natural preservation area located in Ilha Solteira, SP, Brazil, that harbors several species of forest fauna, including capybaras. It was observed that Australis and Tarassovi were present in the more reactive rats (15.4 %). Silva [26] found 33.3 % positivity among rodents captured in the zoo in Ribeirão Preto, SP, Brazil, which were reactive to the serovars Copenhageni and Pyrogenes.

Incidental infections caused by strains carried by other domestic and free-living animals are dependent on environmental and management factors, which results in direct and indirect contact of the animal with the urine of reservoirs of the bacterium [31].

The association with the most frequent serovar and their most frequent maintenance host is very important in leptospirosis. The transmission among maintenance hosts can involve contact with infected urine and other secretions, like placental fluids or can be venereally transmitted. There is a possible association with serological response from wild animals and the maintenance hosts: for serovar Icterohaemorrhagiae, rats are the common maintenance hosts; for pigs, cattle, opossums and skanks, serovar Pomona; and for raccoons, muskrats, skunks and voles, serovar Grippotyphosa [32].

The diversity of reagent serovars in this study suggests the previous contact of capybaras with other wild and domestic animals, including synantropic rodents. The fact that one species that carries *Leptospira* cohabitates with another, helps in the dissemination of the pathogen within species, which is one of the causes of the presence of different serovar strains in an infected animal.

## Conclusions

Despite the positive results regarding the serological results, new studies must be performed with a higher number of animals from different regions to assess the importance of leptospirosis in capybaras. In addition, isolation of the agent in urine, for example, should be carried out in order to evaluate the real importance of leptospirosis in capybaras, to consequently verify the zoonotic potential of this species as source of infection to humans and other animals.

## Ethics approval

This study was aproved by the Ethics Committee on Animal Use (CEUA) of São Paulo State University (UNESP) under registration no. 148–2007.
